# Sexual behavior experiences and characteristics of male-female partnerships among HIV positive adolescent girls and young women: Qualitative findings from Zimbabwe

**DOI:** 10.1371/journal.pone.0194732

**Published:** 2018-03-22

**Authors:** Webster Mavhu, Elizabeth Rowley, Ibou Thior, Natalie Kruse-Levy, Owen Mugurungi, Getrude Ncube, Suzanne Leclerc-Madlala

**Affiliations:** 1 Centre for Sexual Health & HIV/AIDS Research (CeSHHAR), Harare, Zimbabwe; 2 Liverpool School of Tropical Medicine, Liverpool, United Kingdom; 3 PATH, Washington, District of Columbia, United States of America; 4 JSI Research & Training Institute, Inc., Arlington, Virginia, United States of America; 5 United States Agency for International Development (USAID), Harare, Zimbabwe; 6 AIDS & TB Unit, Ministry of Health & Child Care, Harare, Zimbabwe; 7 United States Agency for International Development (USAID), Arlington, Virginia, United States of America; International AIDS Vaccine Initiative, UNITED STATES

## Abstract

**Background:**

New HIV infections among sub-Saharan Africa's adolescent girls and young women (AGYW, ages 15–24) greatly exceed those of their male peers. In addition, AGYW tend to acquire HIV at a much earlier age. Understanding the factors associated with HIV infection in AGYW could inform effective prevention and treatment interventions for these populations and their male sexual partners.

**Methods:**

This qualitative study, conducted October-November 2016, was a follow on to a quantitative survey that sought to characterize male sexual partners and sexual behaviors of sexually active HIV positive AGYW in Zimbabwe. The qualitative study explored sexual behavior experiences and characteristics of male-female partnerships among the same participants. We conducted in-depth interviews with purposively sampled AGYW (16–24 years). Audio recorded qualitative data were transcribed, translated into English, and thematically coded using NVivo.

**Results:**

28 AGYW (n = 14 urban, n = 14 rural) took part in the in-depth interviews. 50% were 16–19 years old. Discussions with 10/11 (91%) AGYW who were reportedly infected through sex suggested that they had acquired HIV from their husbands or romantic partners. Accounts also suggested that the age difference between respondents and their male sexual partners was ≥5 years. Overall, respondents described two types of male partners: those older (''sugar daddies'', men ≥35 years old) and younger (<35 years). Respondents felt unable to suggest condom use to both older and younger partners. Evident in respondents' accounts was a general low HIV risk perception, particularly with younger men, which was largely due to poor HIV knowledge. Discussions suggested that an AGYW's relationship with either male partner was characterized by some form of violence.

**Conclusions:**

Discussions highlighted the nature and characteristics of relationships between AGYW and their male sexual partners. Findings could inform interventions to engender risk perception among AGYW, promote female-controlled HIV prevention efforts and, foster risk-reduction among men.

## Introduction

In sub-Saharan Africa (SSA), new HIV infections among adolescent girls and young women (AGYW, ages 15–24) remain exceptionally high [1–3]. For example, in South Africa and Zimbabwe, HIV incidence in AGYW is four times higher and twice as high, respectively, as in men of the same age [2]. Additionally, AGYW in SSA tend to acquire HIV infection at a much earlier age compared to their male peers [3–6]. Although the cause of this vulnerability has not been fully elucidated, it is now well-recognized that it is compounded by a range of biological, social and structural factors [2, 3, 6–9].

In addition to the recognition that more effective prevention interventions for AGYW are urgently needed, there is consensus that these must be founded on a better understanding of the range of factors associated with HIV infection in these populations [1–3, 6]. Indeed, one factor that most likely explains SSA AGYW's vulnerability is having sexual relationships with men who are at least five years older than them, and who may have recently acquired HIV or who are already living with HIV but are not on antiretroviral therapy (ART) [3, 5, 10]. An in-depth understanding of the drivers of this partnering pattern, and learning more about these male-female partnerships, is critical for addressing the prevention needs of AGYW [3, 6].

There is currently scant literature on sexual behaviors and male-female partnerships of HIV positive AGYW in SSA in general and Zimbabwe, in particular. We report on a qualitative study that explored in-depth sexual behavior experiences and characteristics of male-female partnerships among sexually active HIV positive AGYW in Zimbabwe. Study findings could inform HIV prevention and treatment interventions for AGYW and their male sexual partners.

## Methods

### Design and setting

This qualitative study was a follow on to a quantitative survey that was conducted with 360 HIV positive AGYW (15–24 years old) recruited from four health facilities offering antiretroviral therapy (ART) and elimination of mother-to-child transmission (EMTCT) services to HIV positive AGYW in Harare, Zimbabwe's capital city, and in two rural facilities in Mazowe district, Mashonaland Central Province. HIV positive AGYW attending these facilities receive ART and adherence support as set out in the prevailing Ministry of Health and Child Care (MoHCC) guidelines [11]. As per MoHCC guidelines, after ART initiation, AGYW are seen monthly, with CD4 monitoring at 6 monthly intervals. Although routine viral load testing was adopted by MoHCC in 2010 and scale-up began in 2012 [12], the MoHCC is currently only able to provide targeted rather than universal viral load testing [13]. We selected Mazowe among rural areas because it is one of the DREAMS (**D**etermined, **R**esilient, **E**mpowered, **A**IDS-free, **M**entored, and **S**afe) focus sites. DREAMS is a public–private partnership that aims to reduce HIV infections among AGYW in 10 SSA countries (Zimbabwe included) through a multipronged approach that includes national campaigns, access to combination prevention, the engagement of men, integration of HIV services into sexual and reproductive services, and efforts to keep girls in school [3].

The quantitative survey sought to characterize male sexual partners and sexual behaviors of sexually active HIV positive AGYW (15–24 years old) in Zimbabwe who became infected either perinatally or through sex. AGYW were recruited into the study if they were aware of their HIV positive status (status confirmed by clinic record and/or enrollment in ART or EMTCT program), and sexually active (had penetrative sex within the last 12 months). This qualitative study explored in-depth, issues that emerged from the quantitative survey. Between October and November 2016, we conducted in-depth interviews with a predetermined number of HIV positive AGYW (n = 28) after informed consent. Purposive sampling ensured a balance between two age groups (16–19 and 20–24 years) as well as place of residence (urban rural). No one declined to take part in the study. The qualitative study sample (n = 28) was deemed sufficient to achieve theme saturation.

### Data collection, processing and analysis

The in-depth interviews took place in a private room or space in all recruiting facilities, and were facilitated by four trained female researchers who had previously administered the quantitative survey. Discussions were informed by a topic guide (S1 File). All discussions were held in Shona, the respondents' language. All discussions were audio-recorded; hand-written notes were taken as back-up. The discussions lasted approximately 1 hour. As per Medical Research Council of Zimbabwe (MRCZ) requirements, each participant was reimbursed US$5 for their time and effort.

Audio-recorded data were transcribed and translated verbatim into English. Interview summaries were then written for each in-depth interview and used to come up with a provisional coding framework. A quarter of the in-depth interviews (n = 7) were then independently coded by two different researchers using the coding framework. Discrepancies were resolved through discussion until consensus was reached. Any additional codes identified from the first set of transcripts were added to the coding framework. Transcripts were entered into NVivo (QSR International, Melbourne, Australia), a qualitative data storage and retrieval program. Transcripts were then coded using the modified coding framework; care was taken to identify any additionally emerging codes. Codes were grouped and emerging themes were then identified using thematic analysis [14].

### Ethical considerations

Ethics approval was given by MRCZ (A/2076) and The John Snow Incorporated Institutional Review Board (#16–033). Written informed consent, including for audio recording, was obtained from all respondents prior to their participation in the study. Additionally, all respondents below the age of 18 provided written informed assent in addition to caregiver consent. Confidentiality assurances were given during the assent/consent process. Names and other personal identifiers were removed from transcripts before they were analyzed.

## Results

### Participant characteristics

Table 1 shows participant characteristics by age category. Twenty-eight in-depth interviews were conducted with HIV positive AGYW (n = 14 urban, n = 14 rural). Half of the participants were 16–19 years old. Among 16–19 year-olds, an equal number perceived that they had either acquired HIV through sex or perinatally. The proportion reporting perinatal rather than sexual infection was, however, higher among 20–24 year-olds (57.1% vs. 35.7%, respectively). Respondents aged 20–24 years also reported a higher number of lifetime sex partners, with one participant estimating a total 20 males. Below, we present the main themes that emerged from discussions with respondents.

**Table 1:** Participant characteristics by age category.

In-depth interviews (AGYW)	16–19 years (N = 14)	20–24 years (N = 14)
*Setting*		
Urban	8 (57.1)	6 (42.9)
Rural	6 (42.9)	8 (57.1)
*Perceived source of HIV infection*		
Heterosexual sex	6 (42.9)	5 (35.7)
Perinatally	6 (42.9)	8 (57.1)
Don't know	2 (14.3)	1 (7.1)
*Number of sex partners*		
Median no. of lifetime sex partners (mix-max)	2 (2–8)	3 (2–20)
Median no. of sex partners in last 12 months (min-max)	1 (1–4)	1 (1–7)

Note: Data are n (%) unless specified otherwise.

### Perceived HIV transmitter, awareness of status and vulnerability

Ten of the 11 AGYW who were reportedly infected through sex thought they had acquired HIV from their husbands or romantic partners. A young woman stated, *'I got HIV from my husband because he was once married to a certain woman'* (rural 21 year-old). Another said, *'I got it [HIV] when I slept [had sex] with the father of my child'* (rural 16 year-old). Consistent with quantitative data, in-depth interviews indicated that the age difference between respondents and their husbands or boyfriends was at least 5 years.

A quarter of respondents reported only becoming aware of their HIV status through HIV testing conducted during antenatal care (ANC). *'I went to "register for ANC"*. *That is when I was tested and told I had HIV'* (rural 17 year-old). For some, awareness of HIV status happened quite late into the pregnancy. *'When I knew I had HIV*…*I went to the hospital when I was pregnant with my second baby*. *I was 6 months pregnant*. *I didn’t know I was positive*. *When I went to the antenatal clinic that is when I was told I was positive [HIV]'* (rural 23 year-old).

Orphanhood and ill-treatment heightened adolescent girls' vulnerability. An adolescent described circumstances leading to her first sex. *'When I first had sex*, *I was 13 years old*. *Then*, *I was staying in the rural areas with my grandmother since my parents had died*. *I would do chores such as cattle herding [mostly done by males]*… *She would abuse me*…*I met a man who promised to take care of me and he impregnated me'* (urban 16 year-old). Vulnerability was sometimes a result of low self-esteem due to HIV-related stigma. *'*…*If one is HIV positive*, *how she is treated matters because if she is looked down upon*, *she ends up thinking*, *"I can’t do this because I am HIV positive"*. *Men take advantage of such girls and impregnate them'* (rural 17 year-old).

### Disclosure of HIV status to sexual partner

A majority of respondents reported that they never disclosed their HIV status to their male sexual partners for a number of reasons, including fear of rejection. *'Maybe you will have found someone you love very much yet he will be HIV negative*. *So you will be afraid of being abandoned*…*'* (urban 16 year-old). All respondents noted that the disclosure process needed to be done cautiously. *'You do not just disclose your status right away*. *You first establish what the person thinks about HIV and how he is going to react and whether he will accept it'* (urban 19 year-old). Some described the consequences of disclosure to a male partner. *'When I was pregnant with my second baby*, *I tested positive*. *He [husband] didn’t sleep in our house that night*. *He just left and never came back'* (rural 24 year-old). Another respondent proclaimed, *'To be honest*, *it is not an easy thing to do*…*He may abandon you and start spreading news about it in the whole neighborhood and you end up feeling out of place because everyone will be talking about you'* (urban 19 year-old). The consequences of disclosure can therefore be far-reaching.

Respondents however articulated the consequences of not disclosing one's status to a male partner including the difficulty of taking ART. *'*…*A problem will arise when she now wants to take her medication [HIV] yet she never disclosed'* (urban 19 year-old). Divorce was also cited as an ultimate consequence of non disclosure. *'Most of the time for one to divorce*, *the husband would have known that she is HIV positive yet she never disclosed to him fearing that she will lose him*. *He will find out through the grapevine*. *People will tell him*, *"The woman you are with is positive‴* (urban 24 year-old). A few reported disclosing their status to avoid future squabbles. *'*…*You would have weighed the options*. *Probably you would have gone through certain experiences where you did not disclose and the end result was not so good*. *So you will just decide to disclose to avoid problems in the future'* (urban 23 year-old).

### Descriptions and perceptions of male sexual partners

Overall, respondents described two types of male partners: those older (''sugar daddies'') and younger. The former were described as men at least 35 years old. *'He [sugar daddy] is much older and can be the same age as your own father'* (rural 23 year-old). Unlike younger men (<35 years), older men (≥35 years) were considered affluent individuals with lots of money, high-paying jobs, cars and businesses. An adolescent girl drew parallels between the two types of men.

Sugar daddy will take you out for surprises like buying you clothes and so on. A younger man usually does not have much money and so he can only do what is worth $2 (USD). For instance, he may buy you "chicken slice" (piece of chicken from fast food outlet). He can't buy you expensive clothes but the sugar daddy buys expensive clothes in addition to paying one's rent. He does everything' (urban 16 year-old).

Due to their relative affluence and hence spending capabilities ("buying power"), older men were naturally attractive to AGYW. Additionally, relationships with older men were sometimes driven by peer pressure. *'If I am in a relationship with a big guy*, *I will tell my friends*, *"These days I am going out with so and so and he is doing this and that for me*. *You see this weave on my head*? *He paid for it*. *You see these shoes*? *He is the one who bought them'* (rural 20 year-old).

### Nature and characteristics of relationships

AGYW reported meeting older men in public places, at functions and entertainment venues and the way the relationship began was almost always informal. *'With sugar daddy*, *he may give you a lift into town*…*He can just stop by himself and offer to take you wherever you are going*. *If going to town for example and then Borrowdale*, *he will end up taking you to Borrowdale where he wasn’t intending to go'* (urban 16 year-old). Relationships with younger men were often established through someone else known to the AGYW. *'It was my sister's husband who when I asked about him*, *told me that he was a nice person*. *When I later asked my sister about him*, *she also said he was an okay guy'* (rural 16 year-old).

Although respondents felt that both types of partners expected sexual favors from the relationship, this was especially so with older men. In fact, all descriptions of relationships with older men highlighted their transactional nature. *'*…*Each time you meet him [sugar daddy]*, *he will be in need of certain "favors" and you will also be in need of a favor*…*It’s a "give and receive" kind of relationship'* (urban 23 year-old). Additionally, although sex with younger men often took place weeks or months from the first meeting, AGYW could have sex with older men the very first time they met. *'The girl will be going wherever and the sugar daddy will be driving his car and if he likes her*, *he hoots and then he asks her to get into his car*. *She is then taken to a lodge and they have sex'* (urban 20 year-old).

Overall, respondents felt unable to suggest condom use to either male partner. Doing so to a younger man would suggest that one had some sexual experience, was of a loose character and therefore an undesirable marriage partner. *'He will think that you were up to no good*, *you were having sex with many people previously*. *It becomes difficult for them to understand you'* (urban 23 year-old). AGYW never attempted to suggest condom use to older men due to among other reasons, age difference and the significant gifts/favors. *'He will want to have unprotected sex*. *He will argue that*, *"I did a lot of things for you and now you want me to use a condom*. *Do you think I am HIV positive*? *You will not fall pregnant‴* (urban 19 year-old). Failure to suggest or use condoms was sometimes due to a lack of knowledge. *'I was 14 years old when I had sex with my first boyfriend*. *We did not use a condom because I knew nothing about condoms'* (rural 17 year-old).

### HIV risk perception

Evident in most respondents' accounts was a general low HIV risk perception, which was largely due to poor HIV knowledge. For example, most AGYW assumed that healthy-looking males could not be HIV positive. *'By merely looking at him*, *nothing seemed to show that he was not healthy*…*I did not get a chance to get tested with him (for HIV) to know his status at that time'* (rural 23 year-old). Others thought that even though a man was HIV positive, one could not easily contract HIV from him. *'It depends on how long they have been sleeping [having sex] because there is a certain number of times*…*You can’t get HIV from sleeping with him just once'* (urban 24 year-old). Yet other respondents reported having unprotected sex (sometimes repeatedly) with men they thought or knew were engaging in risky sexual behavior. In most cases this was due to economically driven desperation.

Interestingly, all respondents (including those that reported heterosexual HIV acquisition) felt that compared to older men, younger men had a lower HIV risk. *'*…*He [sugar daddy] has many girlfriends so the adolescent girl will get HIV from him*. *She* ***might*** *get it from a younger guy but the chances are very slim'* (urban 17 year-old). Paradoxically, those that reportedly acquired HIV through sex cited their husbands or romantic partners—males much younger than the supposed sugar daddies—as sources of their infection. This is once again, testimony to the AGYW's low HIV risk perception.

### Violence to AGYW

Respondents described violence from at least three perpetrators: older man, his wife and a younger man. Describing an older man's violence, a young woman stated, *'What happens mostly is that an older man does not want to know that you are dating another guy*…*Older men are very jealousy such that if he gets to know that you are "double crossing" him*, *he will beat you up because he will be giving you all that you ask for*…*'* (urban 24 year-old). Respondents also mentioned possible violence from an older man's wife after becoming aware of the relationship between the man and the AGYW. Finally, respondents noted the following about a younger man. *'If you are in a relationship with him and at the same time you have a sugar daddy*, *he will beat you up*… *If the younger guy finds out*, *he will be furious that he is planning for the future and you are busy with sugar daddies*. *He will definitely beat you up'* (urban 23 year-old). Another stated *'*…*Even if you report to the police*, *it can be taken as a love affair'* (urban 18 year-old). Noticeable in this assertion is the reluctance to report some of the violence perpetrated by male partners, in part due to perceived poor enforcement of the law.

## Discussion

This study explored sexual behavior experiences and characteristics of male-female partnerships among sexually active HIV positive AGYW.

Accounts of AGYW who reported being infected through sex suggested that they had acquired HIV from their husbands or romantic partners. These findings show once again, that early marriage is a risk factor for HIV acquisition among females in sub-Saharan Africa [15, 16]. Interventions such as DREAMS which aim to keep girls in school [3], thereby averting early marriages should continue to be prioritized. Interventions need to especially target vulnerable AGYW including orphans. Additionally, AGYW perceived men below 35 years of age to pose less HIV risk compared to their older counterparts. Even though our study showed that AGYW have low HIV risk perception with younger partners, other studies have shown that having a partner five years or older increases one’s risk of having sex with an HIV positive man by three times [17]. AGYW need to be taught that a five year age difference between them and their sexual partner is an enough HIV risk factor [3, 5, 10, 17, 18].

Although AGYW perceived HIV risk to be higher in older men, they still had unprotected sex with them suggesting that economic incentives outweighed HIV risk. Moreover, some respondents reported sexual relationships with men they thought or knew had high HIV risk due to economic reasons. These findings not only highlight AGYW's vulnerability but also the fact that economically empowering this group of women should indeed be prioritized as part of the HIV combination prevention package [19]. Evident in most accounts was the persistent lack of empowerment in condom negotiation among AGYW–despite many years of programming for condoms. Over and above intensifying interventions to engender risk perception among AGYW, initiatives need to expand access to and promote female-controlled HIV prevention efforts such as vaginal microbicide ring [20] and pre-exposure prophylaxis [21].

A quantitative survey among the same study participants showed that mean age of learning HIV status was 17 years. This was corroborated by qualitative findings which indicated that most 18–24 year-olds became aware of their status when they were at least 17 years old and already sexually active. Given the benefits of early access to HIV services, innovative approaches such as HIV self-testing, currently being scaled-up in SSA [22, 23], need to be intensified as they have the potential for getting large numbers of adolescents to test for HIV. Additionally, interventions should emphasize the need for especially AGYW to access HIV counseling and testing services before both marriage and conception. Further, AGYW mentioned that they generally do not disclose their HIV positive status to their male sexual partners. Given the potential individual and public health benefits associated with HIV disclosure including improved engagement in care [24–26] and reduced levels of unprotected sexual activity [25, 27, 28], interventions need to encourage and empower HIV positive individuals to disclose to their sexual partners [29]. However, a key consideration will be how to ensure the disclosure process does not result in undesirable consequences.

AGYW reported high levels of violence. As in the rest of SSA, pervasive gender norms and behaviors, coupled with suboptimal knowledge, are the key drivers of sexual and gender-based violence whilst the latter is in itself a key driver of the HIV epidemic [3, 30, 31]. The need to tackle gender-based violence can therefore never be overemphasized. Voluntary medical male circumcision (VMMC) programs, currently being implemented in over a dozen African countries for HIV prevention, provide a unique opportunity to promote positive masculinity and specifically address issues related to gender-based violence and rape [32]. Furthermore, since study findings suggested that men generally have unprotected sex with or without knowledge of their female partners' HIV positive status, interventions targeting men should continue to promote risk-reduction interventions targeting men including condom use, HIV testing services and VMMC.

In conclusion, this qualitative study adds to the scant literature on sexual behaviors and male-female partnerships of HIV positive AGYW in SSA. It enabled an in-depth understanding of the context within which some of the issues identified earlier (through quantitative survey) occurred. Understanding context is key to identification and development of specific interventions. That there was some concordance between quantitative and qualitative findings increases our confidence in the latter's veracity. However, as with all reported data, our findings are potentially prone to social desirability bias–the tendency to provide responses thought to be more favorable or acceptable as opposed to being reflective of true thoughts or feelings [33]. Additionally, we did not interview male partners of AGYW due to concerns around possible accidental HIV status disclosure and other harms. Nonetheless, we believe that the information we obtained is still useful for informing intervention and prevention activities for AGYW and their male sexual partners. Lastly, study findings point to the need for social behavioral research that explores gender power relations and cultural norms as these have a big influence on sexual relationships, gender-based violence and HIV status disclosure.

## Supporting information

S1 File AGYW study ID guide_Oct 2016 eng.docx. (DOCX)

## References

[1] UNAIDS. HIV prevention among adolescent girls and young women: Putting HIV prevention among adolescent girls and young women on the Fast-Track and engaging men and boys Geneva: UNAIDS, 2016.

[2] Schaefer R, Gregson S, Eaton JW, Mugurungi O, Rhead R, Takaruza A, et al Age-disparate relationships and HIV incidence in adolescent girls and young women: evidence from Zimbabwe. AIDS. 2017;31(10):1461–70. Epub 2017/04/21. doi: 10.1097/QAD.0000000000001506 ; PubMed Central PMCID: PMCPMC5457819. 28426534 10.1097/QAD.0000000000001506 PMC5457819

[3] Abdool Karim Q, Baxter C, Birx D. Prevention of HIV in Adolescent Girls and Young Women: Key to an AIDS-Free Generation. J Acquir Immune Defic Syndr. 2017;75 Suppl 1:S17–S26. Epub 2017/04/12. doi: 10.1097/QAI.0000000000001316 . 28398993 10.1097/QAI.0000000000001316

[4] Abdool Karim Q, Abdool Karim SS, Singh B, Short R, Ngxongo S. Seroprevalence of HIV infection in rural South Africa. AIDS. 1992;6(12):1535–9. Epub 1992/12/01. . 1492937 10.1097/00002030-199212000-00018

[5] Gregson S, Nyamukapa CA, Garnett GP, Mason PR, Zhuwau T, Carael M, et al Sexual mixing patterns and sex-differentials in teenage exposure to HIV infection in rural Zimbabwe. Lancet. 2002;359(9321):1896–903. Epub 2002/06/12. doi: 10.1016/S0140-6736(02)08780-9 . 12057552 10.1016/S0140-6736(02)08780-9

[6] Dellar RC, Dlamini S, Karim QA. Adolescent girls and young women: key populations for HIV epidemic control. J Int AIDS Soc. 2015;18(2 Suppl 1):19408 Epub 2015/03/01. doi: 10.7448/IAS.18.2.19408 ; PubMed Central PMCID: PMCPMC4344544. 25724504 10.7448/IAS.18.2.19408 PMC4344544

[7] Harrison A, Colvin CJ, Kuo C, Swartz A, Lurie M. Sustained High HIV Incidence in Young Women in Southern Africa: Social, Behavioral, and Structural Factors and Emerging Intervention Approaches. Curr HIV/AIDS Rep. 2015;12(2):207–15. Epub 2015/04/10. doi: 10.1007/s11904-015-0261-0 ; PubMed Central PMCID: PMCPMC4430426. 25855338 10.1007/s11904-015-0261-0 PMC4430426

[8] Idele P, Gillespie A, Porth T, Suzuki C, Mahy M, Kasedde S, et al Epidemiology of HIV and AIDS among adolescents: current status, inequities, and data gaps. J Acquir Immune Defic Syndr. 2014;66 Suppl 2:S144–53. Epub 2014/06/12. doi: 10.1097/QAI.0000000000000176 . 24918590 10.1097/QAI.0000000000000176

[9] Naicker N, Kharsany AB, Werner L, van Loggerenberg F, Mlisana K, Garrett N, et al Risk Factors for HIV Acquisition in High Risk Women in a Generalised Epidemic Setting. AIDS Behav. 2015;19(7):1305–16. Epub 2015/02/11. doi: 10.1007/s10461-015-1002-5 ; PubMed Central PMCID: PMCPMC4506252. 25662962 10.1007/s10461-015-1002-5 PMC4506252

[10] Kelly RJ, Gray RH, Sewankambo NK, Serwadda D, Wabwire-Mangen F, Lutalo T, et al Age differences in sexual partners and risk of HIV-1 infection in rural Uganda. J Acquir Immune Defic Syndr. 2003;32(4):446–51. Epub 2003/03/18. . 12640205 10.1097/00126334-200304010-00016

[11] MOHCC. Guidelines for Antiretroviral Therapy for the Prevention and Treatment of HIV in Zimbabwe Harare: Ministry of Health and Child Care, 2016.

[12] Bygrave H, editor Scaling up routine viral load monitoring in resource limited settings: field experience from antiretroviral therapy (ART) programmes in rural Zimbabwe and Malawi. 17th International Conference on AIDS and STIs in Africa; 2913; Cape Town.

[13] Mavhu W, Willis N, Mufuka J, Mangenah C, Mvududu K, Bernays S, et al Evaluating a multi-component, community-based program to improve adherence and retention in care among adolescents living with HIV in Zimbabwe: study protocol for a cluster randomized controlled trial. Trials. 2017;18(1):478 Epub 2017/10/21. doi: 10.1186/s13063-017-2198-7 ; PubMed Central PMCID: PMCPMC5649065. 29052529 10.1186/s13063-017-2198-7 PMC5649065

[14] Braun V, Clarke V. Using thematic analysis in psychology. Qualitative Research in Psychology 2006;3(2):77–101.

[15] Mkandawire-Valhmu L, Wendland C, Stevens PE, Kako PM, Dressel A, J. K. Marriage as a risk factor for HIV: learning from the experiences of HIV-infected women in Malawi. Glob Public Health. 2013;8(2):187–201. doi: 10.1080/17441692.2012.761261 23350930 10.1080/17441692.2012.761261

[16] Pascoe SJ, Langhaug LF, Mavhu W, Hargreaves J, Jaffar S, Hayes R, et al Poverty, food insufficiency and HIV infection and sexual behaviour among young rural Zimbabwean women. PLoS One. 2015;10(1):e0115290 Epub 2015/01/28. doi: 10.1371/journal.pone.0115290 PONE-D-14-17367 [pii]. ; PubMed Central PMCID: PMC4307980. 25625868 10.1371/journal.pone.0115290 PMC4307980

[17] Maughan-Brown B, Kenyon C, Lurie MN. Partner age differences and concurrency in South Africa: Implications for HIV-infection risk among young women. AIDS Behav. 2014;18(12):2469–76. Epub 2014/07/23. doi: 10.1007/s10461-014-0828-6 ; PubMed Central PMCID: PMC4451824. 25047687 10.1007/s10461-014-0828-6 PMC4451824

[18] MacPhail C, Williams BG, Campbell C. Relative risk of HIV infection among young men and women in a South African township. Int J STD AIDS. 2002;13(5):331–42. Epub 2002/04/26. doi: 10.1258/0956462021925162 . 11972938 10.1258/0956462021925162

[19] UNAIDS. Prevention Gap Report Geneva: UNAIDS, 2016.

[20] Malcolm RK, Fetherston SM, McCoy CF, Boyd P, Major I. Vaginal rings for delivery of HIV microbicides. Int J Womens Health. 2012;4:595–605. Epub 2012/12/04. doi: 10.2147/IJWH.S36282 ; PubMed Central PMCID: PMCPMC3508658. 23204872 10.2147/IJWH.S36282 PMC3508658

[21] McCormack S, Dunn DT, Desai M, Dolling DI, Gafos M, Gilson R, et al Pre-exposure prophylaxis to prevent the acquisition of HIV-1 infection (PROUD): effectiveness results from the pilot phase of a pragmatic open-label randomised trial. Lancet. 2016;387(10013):53–60. Epub 2015/09/14. doi: 10.1016/S0140-6736(15)00056-2 ; PubMed Central PMCID: PMCPMC4700047. 26364263 10.1016/S0140-6736(15)00056-2 PMC4700047

[22] Harichund C, Moshabela M. Acceptability of HIV Self-Testing in Sub-Saharan Africa: Scoping Study. AIDS Behav. 2017 Epub 2017/07/13. doi: 10.1007/s10461-017-1848-9 . 28699017 10.1007/s10461-017-1848-9 PMC5764831

[23] Indravudh PP, Sibanda EL, d'Elbée M, Kumwenda MK, Ringwald B, Maringwa G, et al ‘I will choose when to test, where I want to test’: investigating young people’s preferences for HIV self-testing in Malawi and Zimbabwe. AIDS. 2017;31(Suppl 3):S203–S12. 28665878 10.1097/QAD.0000000000001516 PMC5497773

[24] Calabrese SK, Martin S, Wolters PL, Toledo-Tamula MA, Brennan TL, Wood LV. Diagnosis disclosure, medication hiding, and medical functioning among perinatally infected, HIV-positive children and adolescents. AIDS Care. 2012;24(9):1092–6. Epub 2012/07/18. doi: 10.1080/09540121.2012.699670 ; PubMed Central PMCID: PMCPMC3428222. 22799616 10.1080/09540121.2012.699670 PMC3428222

[25] King R, Katuntu D, Lifshay J, Packel L, Batamwita R, Nakayiwa S, et al Processes and outcomes of HIV serostatus disclosure to sexual partners among people living with HIV in Uganda. AIDS Behav. 2008;12(2):232–43. Epub 2007/09/11. doi: 10.1007/s10461-007-9307-7 . 17828450 10.1007/s10461-007-9307-7

[26] Spangler SA, Onono M, Bukusi EA, Cohen CR, Turan JM. HIV-positive status disclosure and use of essential PMTCT and maternal health services in rural Kenya. J Acquir Immune Defic Syndr. 2014;67 Suppl 4:S235–42. Epub 2014/12/02. doi: 10.1097/QAI.0000000000000376 ; PubMed Central PMCID: PMCPMC4251910. 25436823 10.1097/QAI.0000000000000376 PMC4251910

[27] Hightow-Weidman LB, Phillips G, 2nd, Outlaw AY, Wohl AR, Fields S, Hildalgo J, et al Patterns of HIV disclosure and condom use among HIV-infected young racial/ethnic minority men who have sex with men. AIDS Behav. 2013;17(1):360–8. Epub 2012/10/12. doi: 10.1007/s10461-012-0331-x . 23054043 10.1007/s10461-012-0331-x

[28] Pinkerton SD, Galletly CL. Reducing HIV transmission risk by increasing serostatus disclosure: a mathematical modeling analysis. AIDS Behav. 2007;11(5):698–705. Epub 2006/11/04. doi: 10.1007/s10461-006-9187-2 ; PubMed Central PMCID: PMCPMC2408867. 17082982 10.1007/s10461-006-9187-2 PMC2408867

[29] Evangeli M, Wroe AL. HIV Disclosure Anxiety: A Systematic Review and Theoretical Synthesis. AIDS Behav. 2017;21(1):1–11. Epub 2016/07/14. doi: 10.1007/s10461-016-1453-3 ; PubMed Central PMCID: PMC5216111. 27406227 10.1007/s10461-016-1453-3 PMC5216111

[30] Ramjee G, Daniels B. Women and HIV in Sub-Saharan Africa. AIDS Res Ther. 2013;10(1):30 Epub 2013/12/18. 1742-6405-10-30 [pii] doi: 10.1186/1742-6405-10-30 ; PubMed Central PMCID: PMC3874682. 24330537 10.1186/1742-6405-10-30 PMC3874682

[31] Sia D, Onadja Y, Hajizadeh M, Heymann SJ, Brewer TF, Nandi A. What explains gender inequalities in HIV/AIDS prevalence in sub-Saharan Africa? Evidence from the demographic and health surveys. BMC Public Health. 2016;16(1):1136 Epub 2016/11/05. doi: 10.1186/s12889-016-3783-5 ; PubMed Central PMCID: PMC5095963. 27809824 10.1186/s12889-016-3783-5 PMC5095963

[32] WHO/UNAIDS. New data on male circumcision and HIV prevention: policy and programme implications Montreux: WHO/UNAIDS, 2007.

[33] Grimm P. Social Desirability Bias. Wiley International Encyclopedia of Marketing 2010.

