# Supplementary material for: Sexual behavior experiences and characteristics of male-female partnerships among HIV positive adolescent girls and young women: Qualitative findings from Zimbabwe
Source: PLoS One. 2018 Mar 22;13(3):e0194732. doi: 10.1371/journal.pone.0194732 (PMC5864257; doi:10.1371/journal.pone.0194732)
Supplement: S1 File — (DOCX) [file pone.0194732.s001.docx]

| **SECTION 1: RESPONDENT DEMOGRAPHICS**  ***I will start by asking some questions about you.*** | | | |
| --- | --- | --- | --- |
| **NO.** | **QUESTIONS AND FILTERS** | **RESPONSES CODES/SKIPS** | |
|  | How old were you at your last birthday? *(If unsure, please give your best guess).* | Years old: |  |
|  | Have you ever attended school?  *ONE RESPONSE ONLY* | No.......................................................................1  Yes......................................................................2 | N🡪F04 |
|  | What is the highest level of schooling you have completed?  *ONE RESPONSE ONLY* | Less than primary...............................................1  Primary...............................................................2  Secondary..........................................................3  Higher than secondary……………….………......4 |  |
|  | Have you ever been married or ever lived with a male as if married?  *ONE RESPONSE ONLY* | No.......................................................................1  Yes......................................................................2 | N🡪F06 |
|  | How old were you when you first got married or lived with someone as if married? *(If unsure, please give your best guess).* | \| Years old: \| \| --- \| |  |
|  | What is your current marital status?  *ONE RESPONSE ONLY* | Married……………………………………………..1  Living together but not married...........................2  Widowed …………………………………………..3  Divorced …………………………………………...4  Separated………………………………………….5  Single.................................................................6 |  |
|  | Are your parents living?  *ONE RESPONSE ONLY* | Both living...........................................................1  Father deceased.................................................2  Mother deceased................................................3  Both deceased....................................................4 |  |
|  | Who do you currently live with?  DON'T PROMPT, CHECK ALL THAT APPLY | No one (I live alone)............................................1  Parent(s).............................................................2  Elder brother/sister..............................................3  Caregiver/relative................................................4  Husband..............................................................5  Boyfriend/romantic partner.....................………..6  Neighbor.............................................................7  Friend/colleague.................................................8  Other (Specify)__________________________9 |  |
|  | Who is the head of your household?  *ONE RESPONSE ONLY* | I am the head of household................................1  Someone else (Specify)____________..............2 |  |
|  | In your view, how did you get HIV?  ***DO NOT PROMPT***  ***ONE RESPONSE ONLY*** | From my mother.................................................1  From a sexual act with someone I knew - We agreed to have sex …………..............................................2  From a sexual act with someone I knew – We did not agree to have sex; used physical force or coercion…..3  From a stranger/rapist.........................................4  From a blood transfusion.....................................5  From a contaminated sharp object......................6  Other (Specify)__________________________7  Don’t know.........................................................98  Declined.............................................................99 |  |
|  | How many people have you had sex with in your **lifetime?** *(If unsure, please give your best guess).*  *Must be completed, given eligibility criteria* | Number of Persons: |  |
|  | How many people have you had sex within the **past 12 months**?  *Must be completed, given eligibility criteria* | Number of Persons: |  |

**HIV DIAGNOSIS AND STATUS**

*As I mentioned earlier, I am going to ask you some questions about HIV. Please remember that anything we talk about will be kept confidential. Please also remember that you do not have to answer any questions that you do not want to answer, but that you should please answer freely and truthfully to the questions you answer. May I continue?*

*Main question*: Can you tell me about when you learned you have HIV?

- How did you find out? Who told you? How old were you when you were told?
- In general, how does the experience of living with HIV influence how most HIV-positive AGYW (15-24 years) think about the future or male relationships?

*Main question*: In general, do you think that others will find out easily that an AGYW is HIV-positive…? If so, how does this usually happen?

- In what ways is this (i.e. others knowing about the HIV-positive status) different for AGYW who became HIV-positive from their mother, compared to AGYW who were infected through sexual contact? Or do you think everyone has the same experience?
- Has it happened to you (i.e. others knowing about your HIV status)? What has it been like?

**TYPES OF MALE PARTNERS**

*Thank you for telling me your thoughts about HIV-positive adolescents and young women (15-24 years of age), and sharing a bit about your experiences. Now, I’d like to ask you some questions about the type of men that AGYW are typically having sex with, especially thinking about the male partners of those who are HIV-positive.*

*Main question*: How would you describe the male partners of HIV-positive AGYW (male partners could be unmarried or married but have one or more additional partners)?

- Are there different types of male partners? What are the names (nicknames) given to these types of male partners?
- How would you describe those different types? What are they like in terms of how they treat their HIV-positive AGYW partner?
- What makes them different from each other (i.e. what is the “typical profile” of each type of male partner…for example, age, occupation, social status, etc.)
- Thinking about these different types of male partners, what makes the type of relationship with each of them to be different? For example, is the relationship with a “blesser” different from a relationship with a “loser” in terms of things like:
  - how partners get to know each other
  - how long the relationship is likely to last. Why?
  - how quickly from the first time they meet are they likely to start sex
  - how frequently the AGYW and male partner see each other
  - where the AGYW and male partner have sex
  - what the AGYW expects from the relationship, the reason she is in the relationship, and whether or not she is likely to be financially dependent on the partner. Why?
  - what the male partner expects from the relationship, and the reason he is in the relationship
  - the type of communication that is possible in that relationship (for example, what kinds of things you can talk about with that partner, or can’t talk about)
  - the feelings of each partner in the relationship, and whether or not there is likely to be any violence (by the male partner against the AGYW)
  - the chances that an AGYW who is not already HIV positive will get HIV from him. Why?

*Main question*: When you think about these different types of male partners, what kinds of differences are there across the different types of partners in terms of gifts and favors? What about…

- The most common types of gifts or favors from that type of partner
- The value of the gifts or favors from that type of partner
- The frequency of gifts or favors from that type of partner
- The sexual expectations that this type of partner has, based on the gifts or favors?
- The dependency of the AGYW on the gifts or favors from that type of partner.
- Many AGYW we talked to said boyfriend/sex is not MAIN source of income - why is this?

*(Note to interviewer: probe to find out the role of the gifts/favors in the motivation for the respondent to be in the relationship – is it important, not very important, etc. and the role of the gifts/favors in maintaining the relationship)*

*Main question*: When you think about these different types of male partners, are there any differences between them in terms of anything to do with sexual health (meaning HIV testing, circumcision, condom use, etc.?) For example – when thinking about these types of partners:

- Which is most likely to be circumcised?
- Which most likely to have ever had an HIV test?
- Which is most likely to have access to condoms? And to use condoms?
- Which is most likely to have multiple sex partners? Which one is likely to have the greatest number of sex partners?

*Main question*: Many HIV-positive AGYW have told us that they feel confident about suggesting to use a condom with a new sex partner. What do you think? What makes it easy or difficult to do this?

- What is the type of relationship when it is easier to do this?
- Who is the type of partner who makes this more/less difficult?
- Have you ever tried to suggest using a condom with a new partner? What was the experience like?

*Main question*: Thinking about the different types of male partners and relationships you have told me about, do you think that an AGYW who has gotten HIV from her mother will be different from an AGYW who has gotten HIV from a sex partner?

- How are these HIV-positive AGYW different from each other in terms of their male partners depending on how they got HIV?
- Are there any differences in male partners or the types of relationships that HIV-positive AGYW have if they have lost their mother, or their father, or both?

**MARRIAGE PARTNERS & DIVORCE**

*Thank you for telling me about these different types of partners that HIV-positive AGYW might have, whether or not they are married. Now I wanted to ask a few questions about the husbands of HIV-positive AGYW.*

*Main question*: Do you think that husbands of most AGYW who are already HIV-positive before marrying are aware of the HIV status of the AGYW? What are some reasons he might know, or might not know? How does he get to know (process). Who initiates discussion?

- We have seen that AGYW ages 20-24 are likely to disclose compared to those 15-19 years old. Why is this?

*Main question*: We’ve seen that among the HIV-positive AGYW we’ve spoken with so far, almost one in five are divorced – within this age group, which males and females do you think are most likely to get divorced? Why? How does the divorce usually happen?

**CURRENT/MOST RECENT MALE PARTNER**

*Now, I’d like to ask you some questions about your current/most recent sexual partner. When I say “sexual partner”, I mean the male with whom you regularly have sex, or the male with whom you have had sex with in the past 12 months even if it is not regular. By sex we mean vaginal or anal. We would like very much to have you help us by telling us as much as you can. Please remember that neither I, nor anyone on the study team, will be judging you as you tell us about your experiences. Remember that your name will never be linked to the information that you give us. May I continue?*

*Main question*: Can you tell me a bit about your relationship with him?

*Note to interviewers*: Use the categories the respondent has identified in earlier questions to probe about the current/most recent type of relationship.

- Is he your husband, or a “spoiler”, or a “loser”, or other type of partner? How would you describe him?
- Is he older than you, or younger than you, or the same age?
- Most AGYW we talked to said they had older male partners. Why is this? Explore differences between same age, older by up to 5 yrs, 5-10 yrs & more than 10 yrs old.
- What does he do? (for example – studying, working, etc. – probe for details, e.g. working in what, studying at what level, etc.)
- What is his level of education?
- How does he get money? What does he use his money for?
- Where does he spend most of his time? What about work time? What about leisure time?
- Who does he spend most of his time with?
- Does he travel around very much? How would you describe his travels – is it all the time or once a month or once every few months? Why does he travel?
- What are his main interests? (Can be anything – family, education, politics, movies, music, sports – anything that he is interested in)
- What word can you think of that best describes him?

*For respondents who are not married to the current/most recent male partners*:

- Does he live close by to you (for example – in the same town/neighborhood) or if not, how far away does he live?
- Who does he live with? Is he married? Does he have children? (if yes, where do they live?)

*Main question*: How did you get to know him?

- How long ago did you first meet?
- Were you and he living in the same town or neighborhood at that time? The same neighborhood where you are living today or somewhere else?
- Did someone introduce you or did he introduce himself to you?
- Where did you meet for the first time? (meaning is this partner someone in the community that you had always known, or someone you met at school, or in town, or at church, or in a bar, or somewhere else?) Is it easier to have sex with someone from same community compare to that from outside? Why?
- HIV-positive AGYW we have been talking to mentioned that they met their **CURRENT** sex partners either at transportation/commercial/public venues or school/church. What are the similarities or differences between partners met at these two type of venues?
- What about the first time you had sex – what was this like?
  - How long had you known each other?
  - Where were you?
  - What did you feel about it?
  - Did he use physical violence to force you to have sex?
  - Did he pressure you to have sex in other ways that were not violent? Can you please tell me about this?

*Main question*: How would you describe your relationship with him?

- Did anyone influence you about being in a relationship with him? Did you seek advice from anyone? (*Note to interviewer: Only ask this question if the respondent does not indicate that it was a forced relationship.)*
- What are all the good things about the relationship? Is there anything that you don’t like? Do you ever think about trying to end the relationship?
- Why do you think he is in the relationship with you?
- What kinds of things can you talk about with him? Is there anything you can’t talk about with him?
  - What about your HIV status? (Is he aware? If yes – how did he come to learn about it? What does he think or say about it? If no - have you tried to discuss it with him? What makes it difficult to discuss with this male partner?)
- Does he give you gifts or favors? If so, can you tell me more?
  - What are these gifts or favors usually?
  - Is it often, or not very often?
  - What do these gifts and favors mean to you, and to the relationship?
- Do you know or think that he has other sex partners now or in the past, since the time you have started to be together?
  - If yes - How do you get to know about this?
  - Can you tell me anything about these other partners?
  - What about you? Do you/Did you have other sex partners at that time? Can you tell me anything about these other partners?
  - Most HIV+ AGYW we have talked to said they knew or thought their partner was having sexual relation with someone else. Why would they still have sex with them?

*Main question*: What can you tell me about his health? Do you think he is healthy? Why?

- Do you know if he has had an HIV test? If yes – did he disclose to you the results? If no – why do you think he has not had an HIV test?
- Is he circumcised? If no – why do you think he has not gotten circumcised? Have you tried to discuss it with him?
- What does he say about using condoms with you?
  - If he has other sex partners, do you think/know if he uses condoms with the other sex partners?
  - If he uses condoms, where does he usually get them?
- Do you ever talk about family planning with him? What does he say about family planning?
- Do you know if he has or has ever had any STIs? If yes, which one(s)? Where do you think he got it/them?
- Dou think that most HIV+ AGYW are able to refer their partners for VMMC, HIV testing, PrEP (if not HIV positive already)? Why or why not? What about you?

**FIRST MALE PARTNER**

*Note to interviewer*: If you have determined that the current male partner is also the respondent’s first male sex partner, you can skip this section and move to the next section.

*Thank you for telling me about your current/most recent sex partner. Now I would like to ask you some questions about the first time that you had sex. By sex we mean vaginal or anal.*

*Main question: What can you tell me about when you had sex for the very first time?*

- About how long ago was this - how old were you?
- What were you doing then – were you studying or working or staying at home?
- Were you already HIV-positive at this time?
- Did you use a condom with this person? *If yes,* did you use it from start to finish? *If no,* what did you do to avoid getting pregnant? *If no,* what was preventing you and him from using a condom? Did you talk about using a condom the first time you had sex – what was that discussion like?

Main question: What can you tell me about the male partner with whom you had sex for the very first time?

(Note to interviewer: try to establish who it was - a friend of the respondent, an older friend of the family, a teacher, someone else in the community, someone she did not know)

*Main question*: Can you tell me about your relationship with him?

*Note to interviewers*: Use the categories the respondent has identified in earlier questions to probe about the current/most recent type of relationship.

- Was he your husband, or a “spoiler”, or a “loser”, or other type of partner?
- How did you get to know him?
- Were you and he living in the same town or neighborhood at that time? The same neighborhood where you are living today or somewhere else?
- Where did you meet for the first time? (meaning is this partner someone in the community that you had always known, or someone you met at school, or in town, or at church, or in a bar, or somewhere else?)
  - Did someone introduce you or did he introduce himself to you?
- What about the first time you had sex – what was this like?
  - How long had you known each other?
  - Where were you?
  - Did he use physical violence to force you to have sex?
  - Did he pressure you to have sex in other ways that were not violent? Can you please tell me about this?
  - What do you think were the reasons that the partner wanted to have sex with you?

*Main question*: How would you describe him?

- Was he older than you, or younger than you, or the same age?
- What did he do? (for example – studying, working, etc. – probe for details – working in what, studying at what level, etc.)
- What was his level of education?
- Was he married? Did he have children? Who was he living with?
- How did he get money?
- Where did he spend most of his time? What about work time? What about leisure time?
- Who did he spend most of his time with?
- Did he travel around very much? How would you describe his travels – was it all the time or once a month or once every few months? Why did he travel?
- What were his main interests? (Can be anything – family, education, politics, movies, music, sports – anything that he is interested in)
- What word can you think of that best describes him?
- **What is the biggest difference between your first and current sex partners?**

*Main question*: Why did you have sex for the very **FIRST TIME** with this person? (i.e. Why did first sex happen with this particular person)?

- What was your motivation for sex? Reasons for engaging in first intercourse?
- Did anyone influence you about being in a relationship with him? Did you seek advice from anyone? (*Note to interviewer: Only ask this question if the respondent does not indicate that it was a forced relationship.)*
- Did you have sex with him just that first time and never again, or were there several instances in the time that followed?
- What about your HIV status? (If you were already HIV-positive, was he aware? If yes – how did he come to learn about it? What did he think or say about it? If no – did you try to discuss it with him?
- Did he give you gifts or favors? If so, can you tell me more?
  - What were these gifts or favors usually?
  - Was it often, or not very often?
  - What did these gifts and favors mean to you, and to the relationship?
- Do you know or think that he had other sex partners at that time?
  - If yes - How do you get to know about this?
  - What did you think about this?
  - Can you tell me anything about these other partners
  - What about you? Did you have other sex partners at that time? Can you tell me anything about these other partners?

*Main question*: What can you tell me about his health? Do you think he was healthy? Why – what makes you think this?

- Do you know if he had ever had an HIV test at that time? If yes – did he disclose to you the results? If no – why do you think he had not had an HIV test?
- Was he circumcised? If no – why do you think he had not gotten circumcised? Did you try to talk with him about it?
- What did he say about using condoms with you?
  - If he had other sex partners, do you think/know if he used condoms with the other sex partners?
  - If he used condoms, where did he usually get them?
- Did you ever talk about family planning with him? What did he say about family planning?
- What about any other STIs *–* do you know if he had any STIs? If yes, which one(s)?

**WRAP-UP QUESTIONS**

*Thank you for helping us to better understand the different types of male partners of HIV-positive adolescents and what these relationships are like. I have a few wrap up questions to learn what you think about the needs of HIV-positive AGYW and male partners.*

*Main question*: What would you say puts AGYW at risk of HIV infection?

- What do you think would reduce the risk of HIV infection among AGYW?
- In your view, what do the male partners of HIV-positive AGYW (including male partners who are HIV-positive, and male partners who are HIV-negative) need (i.e. interventions) in order to prevent the spread of HIV?

*Main question*: Is there anything else you want to tell me about adolescent girls and young women and their partners?
